# Telehealth to support referral management in a universal health system: a before-and-after study

**DOI:** 10.1186/s12913-021-07028-5

**Published:** 2021-09-25

**Authors:** Sabrina Dalbosco Gadenz, Josué Basso, Patrícia Roberta Berithe Pedrosa de Oliviera, Stephan Sperling, Marcus Vinicius Dutra Zuanazzi, Gabriel Gausmann Oliveira, Ivonice Martins da Silva, Raphael Mendes Motta, Luana Gonçalves Gehres, Érica de Brito Mallmann, Átila Szczecinski Rodrigues, Daniela V Pachito, Beatriz de Faria Leao

**Affiliations:** 1 Regula Mais Brasil Hospital Sírio-Libanês, Rua Barata Ribeiro,142, 01308-000 São Paulo, SP Brazil; 2 Ministry of Health, Esplanada dos Ministérios, Bloco G, 3º Andar, 70058-900 Brasília, Distrito Federal, Brazil

**Keywords:** Referral and Consultation, Telemedicine, e-Health, Telehealth, Delivery of Health Care, Integrated

## Abstract

**Background:**

Management of patient flow within a healthcare network, allowing equitable and qualified access to healthcare, is a major challenge for universal health systems. Implementation of telehealth strategies to support referral management has been shown to increase primary care resolution and to promote coordination of care. The objective of this study was to assess the impact of telehealth strategies on waiting lists and waiting times for specialized care in Brazil.

**Methods:**

Before-and-after study with measures obtained between January 2019 and February 2020. Baseline measurements of waiting lists were obtained immediately before the implementation of a remotely operated referral management system. Post-interventional measurements were obtained monthly, up to six months after the beginning of operation. Data was extracted from the database of the project. General linear models were applied to assess interaction of locality and time over number of cases on waiting lists and waiting times.

**Results:**

At baseline, the median number of cases on waiting lists ranged from 2961 to 12,305 cases. Reductions of the number of cases on waiting lists after six months of operation were observed in all localities. The magnitude of the reduction ranged from 54.67 to 88.97 %. Interaction of time measurements was statistically significant from the second month onward. Median waiting times ranged from 159 to 241 days at baseline. After six months, there was a decrease of 100 and 114 waiting days in two localities, respectively, with reduction of waiting times only for high-risk cases in the third locality.

**Conclusions:**

Adoption of telehealth strategies resulted in the reduction of number of cases on waiting lists. Results were consistent across localities, suggesting that telehealth interventions are viable in diverse settings.

**Supplementary Information:**

The online version contains supplementary material available at 10.1186/s12913-021-07028-5.

## Background

Established by the 1988 Constitution of Brazil, the Unified Health System (*Sistema Único de Saúde* or SUS) has a landmark in Article 198: “public health actions and services are part of a regionalized and hierarchical network and constitute a single system (…)” [1]. The Organic Law, which regulates the constitutional provisions, was published two years later, on September 1990, Law nº 8080 [2]. In December of the same year, the Presidency of the Republic promulgated the Law nº 8142, a milestone that provides instruments for social control and community participation in the organizational context of the Health System and intergovernmental transfers of financial resources [3]. However, over the last 30 years of providing universal care and managing the navigation of cases through its healthcare networks, SUS has faced a series of challenges, sometimes imposed by the financing model of public health services, and in other occasions imposed by the geodemographic conflicts of the country´s continental dimensions [4–6].

SUS is an integrated system comprised by municipal, state and federal levels, organized in a regionalized and hierarchical network of health actions and services of three levels of complexity [7, 8]. Primary Health Care (PHC) represents a wide basis of this pyramid and is responsible for the coordination of care and organization of access to other levels of the health system [9]. The secondary level is composed of actions and services whose complexity requires specialized professionals and the use of technological resources to support diagnosis and treatment [10]. The third complexity level consists of a set of resources that, in the context of SUS, are related to high technology or high cost, aiming at providing the population with access to qualified services and integrated care. The development of regionalization and hierarchization is thus a fundamental point to achieve coordination and integrality of care, as recommended by the Federal Constitution [10].

In 2008, in order to organize the user flow within the healthcare network and to provide equitable, comprehensive and qualified access to health services, the Ministry of Health of Brazil launched a national policy to establish directives to coordinate the process of referral and counter referral from primary care patients to other levels of care [11, 12].

Few years before the publication of these directives, the Information Technology department of SUS had made available, free of charge to cities and states, a software tool named SISREG to allow for better control of patient flow and to optimize the use of resources, as well as to monitor and evaluate the various processes of referral management. SISREG is currently on its third version with the following main functionalities: planning and distribution of assistance resources on an equal basis, respecting intermunicipal agreements; systematic monitoring of the agreed ceilings between municipalities; organization of referrals at all levels of care; identification of areas of disproportion between demand and offer; and monitorization of executed actions by provider [13].

Regional centers responsible for referral management have their own protocols to prioritize cases on waiting lists in detriment to a simple *first come, first served* model [14]. Regional experiences include the promotion of the coordination of care by PHC providers, who act as gatekeepers to specialized care [15]. Even provided these measures, in several localities the *first come, first served* model still predominates, making the flow of patients possibly unfair and time-consuming. Previous studies suggest that a high proportion of referrals to specialized care could be properly managed at the primary care level, which reinforces the need of effective strategies to manage referrals to specialized care [16–18].

The *Regula Mais Brasil* project aims at employing telehealth strategies to remotely support the regional centers in managing the referral of cases from PHC to specialized care. The integration of telehealth into the referral process to specialized care increases PHC’s resolution; favors coordination of care; promotes therapeutic adherence; decreases readmissions; and encourages quaternary prevention [19–22]. The objective of this study is to assess the impact of *Regula Mais Brasil* project on waiting lists and waiting times to specialized care in localities with a diversity of socioeconomic and demographic characteristics in Brazil.

## Methods

The *Regula Mais Brasil* project was instituted to support the referral of cases from PHC units to specialized care in the universal health system in Brazil. This before-and-after study was conducted within the context of the project, with measures obtained before and after the implementation of telehealth interventions.

The study report was developed in a way to address all items applicable to before-and-after studies of The Strengthening the Reporting of Observational Studies in Epidemiology (STROBE) Statement [23].

Procedures performed were in accordance with the ethical standards of the declaration of Helsinki. The research protocol was approved by the institutional Research Ethics Committee of Hospital Sírio-Libanês (Comitê de Ética em Pesquisa - CEPesq), under the number 28453420.5.0000.5461. Informed consent was waived by the CEPesq, Hospital Sírio-Libanês, considering the focus on the management of case referrals using data from the referral system, and that no type of intervention directed to patients was applied and data was analyzed and presented in an aggregated and unidentified way.

### Implementation of the remotely operated referral management system

The first step for the implementation of remotely operated referral management system was the assessment of the referral management system that was in place in the locality at the moment of the implementation. The maturity of the referral management system was assessed by considering the digital resources available for the review of referrals, including the use of telehealth and other information and communication technology tools, such as teleconsulting; and the appropriateness of the clinical assessment. Many referral management systems did not have their waiting lists under the supervision of a physician and operated entirely on automatic processes, on a ‘first come, first served’ basis. Some referral management systems, likewise, did not have waiting lists digitized, causing loss of follow-up of cases, configuring an even more challenging scenario.

**Fig. 1:**
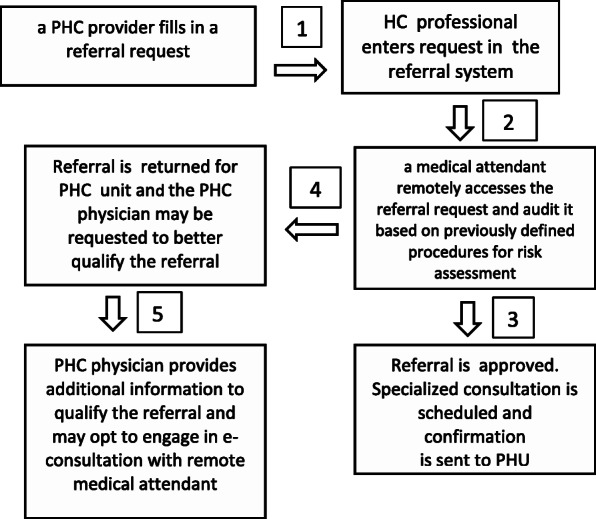
Flowchart depicting the referral assessment

The process employed for managing referrals within the project includes the steps as follow: (i) a PHC provider fills in a referral request; (ii) a healthcare professional from the same PHC unit enters this request in the referral management system; (iii) a medical attendant of the project remotely accesses the referral request and audit it based on previously defined procedures for risk assessment [2]; (iv) this medical attendant approves or returns the referral to the PHC unit; (v) in case of return, the PHC physician may be requested to better qualify the referral, by providing additional information necessary for the adequate prioritization and final decision on the referral. Additionally, PHC physicians may opt to engage in an e-consultation with the remote medical attendant for improved clinical management [24] (Fig. 1).

E-consultation involves the discussion of clinical aspects of cases on waiting lists, between the PHC provider and the medical attendant of the project. Through a toll-free number made available by the Ministry of Health, cases are discussed considering the best clinical evidence, individualized needs and available local resources. The main objective is to define the need and priority of the referral or to guide the case for follow-up in primary care. In this way, e-consultation strengthens quaternary prevention and the resoluteness of primary care. Information related to consultation is permanently recorded in the referral management system in case the PHC provider needs to review it.

*Regula Mais Brasil* has operational units in Sao Paulo, Porto Alegre and Distrito Federal. These three units are responsible for managing referrals from PHC units to specialized care in four localities, namely, the cities of Porto Alegre, Belo Horizonte, the state of Amazonas and the Distrito Federal (federal district). These sites were chosen to be included in the project to reflect the socioeconomical and demographic diversity observed in Brazilian geographies.

### Data analyses

Baseline measurements of the number of cases on waiting lists of medical specialties and times to specialized care were obtained immediately before the implementation of the telehealth regulation intervention (T0). Post-interventional measurements were obtained monthly up to six months after the beginning of operation (T1 to T6).

Waiting lists of each medical specialty were expressed in number of cases waiting for a specialized service. To be included in the analysis, a waiting list must have attended the following eligibility criteria: (i) minimum operating time of six months; (ii) minimum of 100 cases in the waiting list at the baseline; and (iii) no change in local policies that could have affect the admission of cases. All waiting lists fulfilling such criteria were included for analysis. Non-eligible waiting lists and reasons for exclusion can be found in the Supplementary material (Table 1, Supplementary material). The period considered for analysis was January 2019 to February 2020. Measurements obtained after this period were not considered for inclusion due to the confounding effects of the Covid-19 pandemic over the number of cases on waiting lists.

**Table 1:** Number of cases waiting for specialized care, by medical specialty

Medical specialty	AM	BH	PA	Total
Cardiology	12,821	-	-	12,821
Endocrinology	5215	-	-	5215
Gastroenterology	10,466	-	392	10,858
Gynecology	-	-	1983	1983
Neurosurgery	-	-	1491	1491
Neurology	-	11,594	3938	15,532
Orthopedics	22,243	-	7161	29,404
Pediatric orthopedics	-	-	801	801
Proctology	1783	-	-	1783
Rheumatology	-	12,640	-	12,640
Urology	8642	12,305	5545	26,492
Vascular surgery	-	-	5849	5849
Total	61,170	36,539	27,160	124,869

*AM* Amazonas; *BH* Belo Horizonte; *PA* Porto Alegre

Changes from baseline of number of cases in waiting lists and waiting times by locality after the implementation of the telehealth strategies were reported. Whenever possible, waiting times for specialized care were analyzed by priority levels. All variables of interest were extracted from the project database.

General mixed models were applied to investigate interaction between time measurements and localities. The choice between the fixed effect model or the random effects model were made by prioritizing the model with lower Akaike’s information criterion (AIC). Statistical analyses were performed in SPSS v22.

## Results

After having assessed the study inclusion criteria, a total of 17 waiting lists and 124,869 cases were included in the analyses. These waiting lists corresponded to medical specialties in Amazonas (61,170 cases), Belo Horizonte (36,539 cases) and Porto Alegre (27,160) (Table 1). Data obtained from Distrito Federal could not be included in analyses due to changes in policies related to the organization of patient flow, ultimately leading to an exclusion of 14,005 cases, which represented 10.08 % of the total number of cases. All included waiting lists were analyzed to assess the number of cases waiting for specialized care before the implementation of the intervention and monthly, over the following six months. Waiting times were assessed for all cases.

**Fig. 2:**
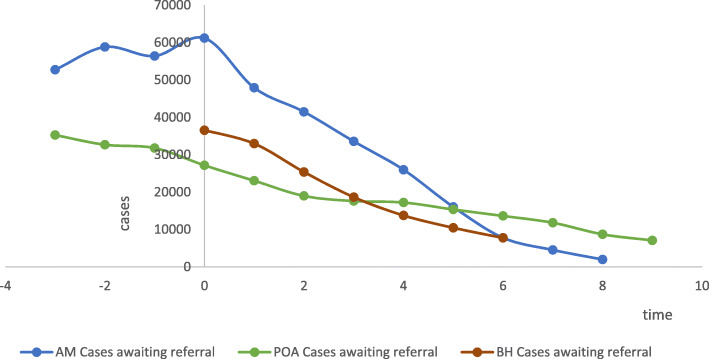
Number of cases waiting for specialized care over time, by locality

Summary measures related to number of cases in waiting lists per locality over, taken at the planned intervals, are presented in Table 2. In all three localities, there was a reduction of the number of cases waiting for specialized care over time (Fig. 2). In Amazonas, the number of cases on waiting lists was reduced by 88.97 % after six months and in Belo Horizonte and Porto Alegre, the reduction was of 76.63 % and 54.67 %, respectively.

**Table 2:** Number of cases on waiting lists over time, by locality

Locality	Summary measure	Time Point						
		0	1	2	3	4	5	6
AM	N of waiting lists	6	6	6	6	6	6	6
	Mean	10195.00	7984.83	6911.00	5593.67	4330.50	2672.67	1298.17
	Median	9554	8510	8264	7610	5319	3521.50	1053.50
	Standard deviation	7072.45	5308.85	5617.23	4351.55	3478.05	2068.74	1249.51
	Minimum	1783	1423	73	48	36	28	45
	Maximum	22243	15059	13973	9197	8254	4469	2971
	Range	20460	13636	13900	9149	8218	4441	2926
	Interquartile range	10819.50	10546.75	11173	9032	7182.25	4335.25	2608
	P value Shapiro-Wilk	0.777	0.710	0.366	0.023	0.200	0.046	0.324
BH	N of waiting lists	3	3	3	3	3	3	3
	Mean	12179.67	10988.33	8459.67	6217.00	4588.00	3488.00	2583.33
	Median	12305	12041	8939	6233	4581	3561	2875
	Standard deviation	534.14	2023.05	1301.00	733.13	153.62	818.94	806.10
	Minimum	11594	8656	6987	5476	4438	2635	1672
	Maximum	12640	12268	9453	6942	4745	4268	3203
	Range	1046	3612	2466	1466	307	1633	1531
	Interquartile range	-	-	-	-	-	-	-
	P value Shapiro-Wilk	0.609	0.107	0.380	0.964	0.925	0.852	0.391
PA	N of waiting lists	8	8	8	8	8	8	8
	Mean	3395	2881	2373.38	2201.88	2152	1918.13	1703.63
	Median	2960.50	2166	1641.50	1540.50	1508.50	1436.50	1342
	Standard deviation	2410.60	2230.52	1754.57	1513.98	1436.76	1413.62	1002.55
	Minimum	392	399	422	618	798	612	671
	Maximum	7161	6600	5332	4782	4816	4546	3296
	Range	6769	6201	4910	4164	4018	3934	2625
	Interquartile range	4799.50	3936.75	3026.50	2682	2409.25	2320.50	1828
	P value Shapiro-Wilk	0.146	0.4419	0.2960	0.2283	0.1226	0.1466	0.1021

*AM* Amazonas; *BH* Belo Horizonte; *PA* Porto Alegre

General mixed model with the fixed effect model did not show statistically significant interactions between localities. There was a consistent interaction between time measures, with statistically significant differences between times from the second month onward (Table 2, Supplementary material).

**Fig. 3:**
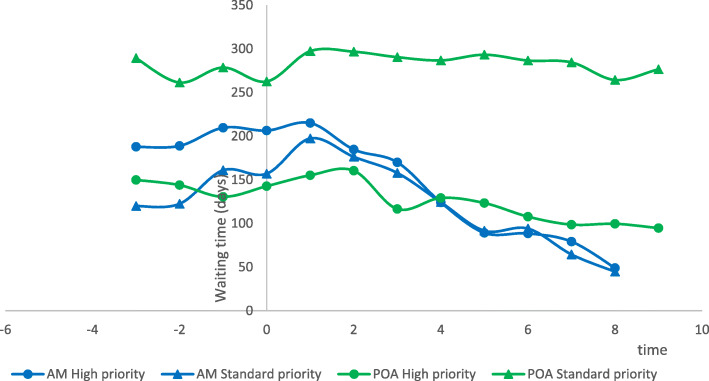
Waiting times for specialized care over time, by priority level

Median waiting times ranged from 159 to 241 days at baseline. After six months of operation, there was a decrease to 59 and 127 waiting days in Amazonas and Belo Horizonte, respectively, with no effect on waiting times in Porto Alegre (Table 3). When cases were separated by priority levels, waiting times showed a reduction for high-priority but not for standard-priority cases in Porto Alegre, and for both levels of priority in Amazonas (Fig. 3).

**Table 3:** Waiting times (in days) over time, by locality

Locality	Summary measure	Time Point						
		1	2	3	4	5	6	7
AM	N	6	6	6	6	6	6	6
	Mean	181.6537	206.1679	180.4773	163.9008	124.1766	90.4063	91.3127
	Median	159	183	172	143	101	70	59
	Std. Deviation	101.1435	107.1458	97.0459	116.0106	101.5863	75.5413	80.0390
	Minimum	73	100	65.0238	31.7356	23.8765	19.3965	18.7829
	Maximum	331	381	304	323	279	207	214
	Range	258	281	239	291	255	188	196
	Interquartile Range	175	186	201	234	192	138	148
	p-value Shapiro-Wilk	0.4744	0.5179	0.5942	0.5730	0.4891	0.3665	0.2016
POA	N	8	8	8	8	8	8	8
	Mean	202.5662	226.1986	228.5259	203.4654	207.9347	208.2794	197.1528
	Median	218	247	265	223	230	226	218
	Std. Deviation	111.1520	111.7048	97.9983	102.0944	98.0885	83.6817	83.6399
	Minimum	56	60	63	62	67	66	62
	Maximum	353	368	333	367	373	308	298
	Range	297	307	270	304	305	242	235
	Interquartile Range	214	212	175	163	141	143	150
	p-value Shapiro-Wilk	0.5719	0.6688	0.3261	0.7676	0.7208	0.6785	0.5031
BH	N	3	3	3	3	3	3	3
	Mean	262.6667	250.3333	252.3333	184.6667	201.6667	200.3333	162.6667
	Median	241	239	232	146	112	138	127
	Std. Deviation	128.8733	155.3104	150.5335	102.6174	161.4074	111.4465	82.5005
	Minimum	146	101	113	107	105	134	104
	Maximum	401	411	412	301	388	329	257
	Range	255	310	299	194	283	195	153
	Interquartile Range	-	-	-	-	-	-	-
	p-value Shapiro-Wilk	0.7209	0.8792	0.7761	0.3651	0.0414	0.0343	0.2671

*AM* Amazonas; *BH* Belo Horizonte; *PA* Porto Alegre

General mixed model with the fixed effect model did not show statistically significant interactions between localities. There was interaction between some time measures adjusted for locality (Table 3, Supplementary material).

## Discussion

Our results showed that the implementation of a centralized, remotely operated referral management system reduced both the number of cases on waiting lists and waiting times of high-risk patients for specialized care, in all analyzed localities. The consistency of results obtained across the localities included in this study is relevant for the interpretation of findings, since it indicates that telehealth interventions may be effective when supporting the referrals of cases from primary to specialized care in diverse settings within a universal health system, regardless of socioeconomic and demographic characteristics of the involved localities.

Brazil is a country with a vast territory marked by socioeconomic disparities. A diversity of decentralized strategies is implemented throughout the country to guide the referral process from primary care. This diversity imposes challenges to regional healthcare leaderships. Baseline measures highlight such heterogeneity: in Porto Alegre, capital of the southernmost Brazilian state, the number of cases on waiting lists were significantly lower (28,772) than the number of cases in Belo Horizonte in the Southeast Region (36,359). By far, the state of Amazonas, in the North Region of Brazil, presented the largest number of baseline cases (61,170). Due to its peculiar geography, it may be difficult to access healthcare services, since it is not uncommon for a patient to have to travel hours or days by boat to attend a specialized consultation [25]. Therefore, it was expected that the largest number of cases in the baseline would be in the Amazonas state. Furthermore, these three localities differ in relation to the proportion of population covered by private health insurance, population density, Gini ratio, human development index and per capita gross domestic product. However, despite all these differences, a reduction in the number of cases was consistently observed over time in all localities.

The pronounced reduction of cases in waiting lists after the implementation of strategies, such as e-consultation between PHC providers and trained family doctors and specialists, reflects the fact that many cases referred to specialized care could be avoided with proper support to PHC providers. Previous studies conducted in Brazil have shown that 20.6 % of referrals to specialized care could be avoided if properly attended at the PHC unit [16]. One can assume that the referral of such cases to specialized care is at some extent responsible for the overburden and long waiting times at the secondary level of care.

In Porto Alegre, the utilization of risk assessments protocols allowed for a significant reduction in waiting times for high-risk patients but not for those at a standard risk. Out of the three locations analyzed, Porto Alegre has the longest experience in using telehealth risk assessment protocols to manage PHC referrals. This may explain why high-risk patients benefited more in the reduction of waiting times for specialized consultations, confirming the usefulness of a protocol-based risk assessment to increase the equity of the system.

In Brazil, pioneering local initiatives involving telehealth strategies to optimize referral management to specialized care have been conducted, such as the *TelessaúdeRS* program and the implementation of teleconsultations in the primary healthcare system in Belo Horizonte [26–28]. These programs include a wide range of interventions, such as telediagnosis and e-consultations [29]. Applied interventions resulted in the reduction of cases in waiting lists and in the waiting time for high-risk cases, as also observed in the present study [27]. The positive results of the *TelessaúdeRS* program ultimately led to the expansion of activities to other regions of the country, originating the *Regula Mais Brasil* project.

There is previous evidence that telehealth strategies, including e-consultations, risk and tele-triage based on risk assessment protocols can reduce waiting lists for referrals to specialized care in countries that do not have universal health systems. In a scoping review on the topic, Caffery et al. concluded that telehealth interventions obviate the need for face-to-face consultations with specialists in 34 to 92 % of cases [30]. However, the model of referral system applied in SUS is probably unique, which hampers direct confrontation of our results with those from studies conducted in other countries.

This study presents several strengths. First, to the best of our knowledge, this is the first study aiming at the assessment of effects related to telehealth in multiple localities in Brazil. We analyzed data of cases waiting for specialized care in multiple medical specialties from two cities and one state, over a period of six months. The number of cases in waiting lists were extracted directly from the databases of the referral management systems, and all cases of waiting lists eligible to inclusion in the study were analyzed.

The impossibility of assessing multiple time points before the implementation of the project precluded a time series analysis and represents one of the limitations of this study. It was not possible to accurately assess the time trend before the start of the study nor to exclude the influence of other concurrent co-interventions that may have led to an overestimation of the intervention impact. Furthermore, it was not possible to include data of one of the localities due to the adopted changes of the referral process, concurrently with the intervention [31]. Finally, we opted to analyze a maximum of a six-month post-intervention period to achieve a higher number of waiting lists. It was not possible to make inferences to whether a more prolonged operation would result in further reduction of the number of cases in waiting lists or a ceiling effect would be reached at some point.

Considering the findings presented and the knowledge generated by previous studies, it is possible to conclude that the adoption of telehealth strategies to assist the referral management of cases from primary to specialized care is an effective intervention, resulting in the reduction of number of cases in waiting lists. The implementation of telehealth in this context was successful in all analyzed localities, suggesting that telehealth can be extended to a diversity of settings within universal health systems. However, it must be considered that, for the successful implementation of a centralized, remotely operated strategy for managing referrals in the whole country, it would be necessary to harmonize procedures across jurisdictions at a minimum. In municipalities with decentralized referrals, such is the case of Rio de Janeiro, where PHC providers have become personally responsible for scheduling procedures and appointments since the Reform in Primary Care [32, 33], procedures would have to be reviewed and redesigned to allow the centralized operation of the referral management system, within a patient-centric approach. This would be a major issue to be overcome in the way of achieving standardized procedures in Brazil.

## Supplementary Information

**Additional file 1: Table S1** Excluded waiting lists with reasons.**Table S2**: General mixed model: number of cases in waiting lists.**Table S3**: General mixed model for waiting times.**Figure S1**: Reduction of number of cases on waiting lists over time

## Data Availability

Public access to the database is closed. Requests for accessing this data should be addressed directly to the Executive Secretariat of the Ministry of Health of Brazil through the following email address gabinete.se@saude.gov.br.

## Abbreviations

PHC: Primary Health Care
SUS: *Sistema Único de Saúde* or Unified Health System
