# Supplementary material for: Telehealth to support referral management in a universal health system: a before-and-after study
Source: BMC Health Serv Res. 2021 Sep 25;21:1012. doi: 10.1186/s12913-021-07028-5 (PMC8467186; doi:10.1186/s12913-021-07028-5)
Supplement: Supplementary file 1 — Additional file 1: Table S1 Excluded waiting lists with reasons.Table S2: General mixed model: number of cases in waiting lists.Table S3: General mixed model for waiting times.Figure S1: Reduction of number of cases on waiting lists over time [file 12913_2021_7028_MOESM1_ESM.docx]

**Telehealth to support referral management in a universal health system: a before-and-after study**

**Supplementary material**

Table 1 Excluded waiting lists with reasons

| Medical specialty | Locality | Reason |
| --- | --- | --- |
|  |  |  |
| Endocrinology | Porto Alegre | Number of cases at baseline fewer than 100 |
|  |  |  |
| Mental health | Porto Alegre | Change in local policies affecting the admission of cases. |
|  |  |  |
| Pediatric mental health | Porto Alegre | Change in local policies affecting the admission of cases. |
| Pneumology | Porto Alegre | Number of cases at baseline fewer than 100 |
|  |  |  |
| Proctology | Porto Alegre | Number of cases at baseline fewer than 100 |

Table 2: General mixed model: number of cases in waiting lists

| Parameter | Estimate | Std. Error | df | t | Sig. | 95% Confidence Interval | |
| --- | --- | --- | --- | --- | --- | --- | --- |
|  |  |  |  |  |  | Lower Bound | Upper Bound |
| Intercept | 12179·6667 | 2659·9150 | 14 | 4·579 | 0·0004 | 6474·7163 | 17884·6171 |
| Amazonas | -1984·6667 | 3257·7173 | 14 | -·609 | 0·5521 | -8971·7754 | 5002·4421 |
| Porto Alegre | -8784·6667 | 3119·0269 | 14 | -2·816 | 0·0137 | -15474·3140 | -2095·0194 |
| Belo Horizonte | 0^b^ | 0 |  |  |  |  |  |
| Time 1 | -1191·3333 | 1028·3223 | 14 | -1·159 | 0·2660 | -3396·8652 | 1014·1985 |
| Time 2 | -3720·0000 | 1090·0366 | 14 | -3·413 | 0·0042 | -6057·8960 | -1382·1040 |
| Time 3 | -5962·6667 | 1639·0626 | 14 | -3·638 | 0·0027 | -9478·1064 | -2447·2270 |
| Time 4 | -7591·6667 | 1840·4516 | 14 | -4·125 | 0·0010 | -11539·0428 | -3644·2904 |
| Time 5 | -8691·6667 | 2049·1420 | 14 | -4·242 | 0·0008 | -13086·6392 | -4296·6941 |
| Time 6 | -9596·333333 | 2265·4548 | 14 | -4·236 | 0·0008 | -14455·2506 | -4737·4160 |
| Baseline | 0^b^ | 0 |  |  |  |  |  |
| Time 1 * Amazonas | -1018·8333 | 1259·4324 | 14 | -·809 | 0·4321 | -3720·0472 | 1682·3805 |
| Time 2 * Amazonas | 436·0000 | 1335·0167 | 14 | ·327 | 0·7488 | -2427·3261 | 3299·3261 |
| Time 3 * Amazonas | 1361·3333 | 2007·4336 | 14 | ·678 | 0·5087 | -2944·1834 | 5666·8501 |
| Time 4 * Amazonas | 1727·1667 | 2254·0837 | 14 | ·766 | 0·4563 | -3107·3621 | 6561·6954 |
| Time 5 * Amazonas | 1169·3333 | 2509·6762 | 14 | ·466 | 0·6484 | -4213·3867 | 6552·0534 |
| Time 6 * Amazonas | 699·5000 | 2774·6042 | 14 | ·252 | 0·8046 | -5251·4340 | 6650·4340 |
| Baseline * Amazonas | 0^b^ | 0 |  |  |  |  |  |
| Time 1 * Porto Alegre | 677·3333 | 1205·8147 | 14 | ·562 | 0·5832 | -1908·8820 | 3263·5487 |
| Time 2 * Porto Alegre | 2698·3750 | 1278·1812 | 14 | 2·111 | 0·0532 | -43·0510 | 5439·8010 |
| Time 3 * Porto Alegre | 4769·5417 | 1921·9713 | 14 | 2·482 | 0·0264 | 647·3232 | 8891·7601 |
| Time 4 * Porto Alegre | 6348·6667 | 2158·1208 | 14 | 2·942 | 0·0107 | 1719·9578 | 10977·3755 |
| Time 5 * Porto Alegre | 7214·7917 | 2402·8320 | 14 | 3·003 | 0·0095 | 2061·2296 | 12368·3538 |
| Time 6 * Porto Alegre | 7904·9583 | 2656·4812 | 14 | 2·976 | 0·0100 | 2207·3728 | 13602·5439 |
| Baseline * Porto Alegre | 0^b^ | 0 |  |  |  |  |  |
| Time 1 * Belo Horizonte | 0^b^ | 0 |  |  |  |  |  |
| Time 2 * Belo Horizonte | 0^b^ | 0 |  |  |  |  |  |
| Time 3 * Belo Horizonte | 0^b^ | 0 |  |  |  |  |  |
| Time 4 * Belo Horizonte | 0^b^ | 0 |  |  |  |  |  |
| Time 5 * Belo Horizonte | 0^b^ | 0 |  |  |  |  |  |
| Time 6 * Belo Horizonte | 0^b^ | 0 |  |  |  |  |  |
| Baseline * Belo Horizonte | 0^b^ | 0 |  |  |  |  |  |

Fixed effect model. b Reference parameter. AM: Amazonas; BH: Belo Horizonte; PA: Porto Alegre.

Table 3: General mixed model for waiting times

| Parameter | Estimate | Std. Error | df | t | Sig. | 95% Confidence Interval | |
| --- | --- | --- | --- | --- | --- | --- | --- |
|  |  |  |  |  |  | Lower Bound | Upper Bound |
| Intercept | 202,5662 | 39,0570 | 14 | 5,1864 | 0,0001 | 118,7973 | 286,3351 |
| Amazonas | -20,9125 | 59,6605 | 14 | -0,3505 | 0,7312 | -148,8716 | 107,0466 |
| Porto Alegre | 60,1005 | 74,7884 | 14 | 0,8036 | 0,4351 | -100,3047 | 220,5057 |
| Belo Horizonte | 0^b^ | 0,0000 |  |  |  |  |  |
| Time 1 | 23,6325 | 6,2852 | 14 | 3,7600 | 0,0021 | 10,1520 | 37,1129 |
| Time 2 | 25,9597 | 10,8805 | 14 | 2,3859 | 0,0317 | 2,6234 | 49,2960 |
| Time 3 | 0,8993 | 12,2124 | 14 | 0,0736 | 0,9423 | -25,2938 | 27,0923 |
| Time 4 | 5,3685 | 17,0806 | 14 | 0,3143 | 0,7579 | -31,2657 | 42,0027 |
| Time 5 | 5,7133 | 16,6241 | 14 | 0,3437 | 0,7362 | -29,9420 | 41,3685 |
| Time 6 | -5,4134 | 19,3679 | 14 | -0,2795 | 0,7839 | -46,9534 | 36,1266 |
| Baseline | 0^b^ | 0,0000 |  |  |  |  |  |
| Time 1 * Amazonas | 0,8818 | 9,6008 | 14 | 0,0918 | 0,9281 | -19,7100 | 21,4735 |
| Time 2 * Amazonas | -27,1361 | 16,6202 | 14 | -1,6327 | 0,1248 | -62,7828 | 8,5107 |
| Time 3 * Amazonas | -18,6522 | 18,6548 | 14 | -0,9999 | 0,3343 | -58,6627 | 21,3584 |
| Time 4 * Amazonas | -62,8455 | 26,0910 | 14 | -2,4087 | 0,0304 | -118,8052 | -6,8859 |
| Time 5 * Amazonas | -96,9606 | 25,3938 | 14 | -3,8183 | 0,0019 | -151,4249 | -42,4963 |
| Time 6 * Amazonas | -84,9276 | 29,5850 | 14 | -2,8706 | 0,0123 | -148,3810 | -21,4742 |
| Baseline * Amazonas | 0^b^ | 0,0000 |  |  |  |  |  |
| Time 1 * Belo Horizonte | -35,9658 | 12,0353 | 14 | -2,9884 | 0,0098 | -61,7789 | -10,1526 |
| Time 2 * Belo Horizonte | -36,2931 | 20,8345 | 14 | -1,7420 | 0,1034 | -80,9786 | 8,3925 |
| Time 3 * Belo Horizonte | -78,8993 | 23,3850 | 14 | -3,3739 | 0,0045 | -129,0551 | -28,7434 |
| Time 4 * Belo Horizonte | -66,3685 | 32,7068 | 14 | -2,0292 | 0,0619 | -136,5176 | 3,7806 |
| Time 5 * Belo Horizonte | -68,0466 | 31,8328 | 14 | -2,1376 | 0,0507 | -136,3212 | 0,2280 |
| Time 6 * Belo Horizonte | -94,5866 | 37,0867 | 14 | -2,5504 | 0,0231 | -174,1297 | -15,0436 |
| Baseline * Belo Horizonte | 0^b^ | 0,0000 |  |  |  |  |  |
| Time 1 * Porto Alegre | 0^b^ | 0,0000 |  |  |  |  |  |
| Time 2 * Porto Alegre | 0^b^ | 0,0000 |  |  |  |  |  |
| Time 3 * Porto Alegre | 0^b^ | 0,0000 |  |  |  |  |  |
| Time 4 * Porto Alegre | 0^b^ | 0,0000 |  |  |  |  |  |
| Time 5 * Porto Alegre | 0^b^ | 0,0000 |  |  |  |  |  |
| Time 6 * Porto Alegre | 0^b^ | 0,0000 |  |  |  |  |  |
| Baseline * Porto Alegre | 0^b^ | 0,0000 |  |  |  |  |  |

Fixed effect model. b Reference parameter. AM: Amazonas; BH: Belo Horizonte; PA: Porto Alegre.

Figure 1: Reduction of number of cases on waiting lists over time


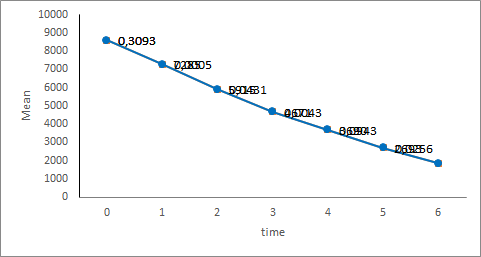


P values between previous time are shown.
